# Inflammatory protein profiles and shunt response in iNPH

**DOI:** 10.1186/s12987-025-00751-9

**Published:** 2026-01-07

**Authors:** Madelene Braun, Maria Ekblom, Eva Freyhult, Mikael Åberg, Dag Nyholm, Kim Kultima, Johan Virhammar

**Affiliations:** 1 Department of Medical Sciences, Neurology, Uppsala University, Uppsala, Sweden; 2 Department of Cell and Molecular Biology, Uppsala University, Uppsala, Sweden; 3 Department of Medical Sciences, SciLifeLab Affinity Proteomics, Uppsala, Sweden; 4 Department of Medical Sciences, Clinical Chemistry, Uppsala University, Uppsala, Sweden

**Keywords:** Normal pressure hydrocephalus, Biomarkers, Cerebrospinal fluid, Neuroinflammation, Outcome, Proteomics

## Abstract

**Introduction:**

Neuroinflammation in the context of idiopathic normal pressure hydrocephalus (iNPH) is poorly studied. Currently, no single objective test can reliably predict outcomes after shunt surgery. The aim was to investigate whether neuroinflammatory proteins in cerebrospinal fluid (CSF) are associated with the characteristic symptoms of iNPH and whether they can predict outcome after shunting.

**Methods:**

Neuroinflammatory proteins were analyzed from preoperative CSF using proximity extension assay (PEA). In total, 92 proteins were analyzed from 74 patients with iNPH referred to shunt surgery at a single center, with follow-up at the same hospital. Symptoms were assessed before surgery and at follow-up (primarily 12 months post-surgery), graded with the Swedish iNPH scale. Associations between protein levels and preoperative symptoms, as well as outcome, were analyzed using linear regression models adjusted for age and sex; outcome models were additionally adjusted for baseline symptom level. Benjamini-Hochberg with a false discovery rate (FDR) of 5% was used to control for multiple analyses.

**Results:**

Of the 92 analyzed proteins, 60 had detectable values greater than 50% and were included in the analyses. No associations between preoperative symptom severity and levels of inflammatory proteins remained statistically significant after correction for multiple comparisons (FDR 5%). After adjustment, CST5 showed a significant negative association with postoperative improvement in balance and continence domains (b = -34, q = 0.017 and b = -35, q = 0.026, respectively) but not for outcome in the total iNPH scale. A general trend was observed where higher levels of inflammatory proteins were linked to less favourable outcomes, although these did not remain statistically significant after correction.

**Conclusion:**

CST5 emerged as the only protein significantly associated with postoperative improvement after shunt surgery, suggesting a potential role in iNPH pathophysiology. Furthermore, no associations were observed between preoperative symptom severity and levels of inflammatory CSF proteins.

**Supplementary Information:**

The online version contains supplementary material available at 10.1186/s12987-025-00751-9.

## Introduction

Idiopathic normal pressure hydrocephalus (iNPH) is characterized by gait disturbance, cognitive impairment, and urinary incontinence. Treatment typically involves ventriculo-peritoneal or, less commonly, lumbo-peritoneal shunt surgery, which alleviates symptoms in 60–80% of patients [1–3]. The most frequently used invasive test to select shunt candidates, the cerebrospinal fluid (CSF) tap test, has high specificity for predicting outcome after shunting, however its limited sensitivity means that some patients with negative results may still benefit from surgery [4, 5]. Consequently, there is a need for additional reliable, objective biomarkers to predict surgical outcomes. However, studies investigating the prognostic value of CSF biomarkers have yielded inconclusive results [6–8].

Many pathological features have been observed in iNPH, including cerebral hypoperfusion, impaired glymphatic function, astrogliosis, neuroinflammation, and blood-brain barrier disruption [9–12]. However, their roles in the pathogenesis of the disease have not been definitively established, and the specific neurochemical changes remain unclear.

Previous studies have shown that CSF in iNPH and other hydrocephalus conditions contains altered levels of inflammatory cytokines and their corresponding receptors, which stimulate immune system activation [13–16]. Downregulation of synaptic inflammatory markers suggests dysfunction of the ependymal layer and may correlate with patient deterioration [17]. Moreover, interleukins (IL) and TNF-α are reported to be involved in the neuroinflammatory process in iNPH, with IL-10 and IL-33 potentially serving as markers for shunt responsiveness [13, 18, 19]. However, reliable predictive CSF biomarkers remain elusive, as those studied show significant variability. Few studies have employed proteomic immunoassay in CSF analysis with a specific aim to identify biomarkers predictive of outcomes in iNPH patients [20].

In this study, we investigated neuroinflammatory proteins in the CSF of iNPH patients using a multiplex proximity extension assay (PEA), aiming to identify associations between inflammatory proteins, preoperative symptoms, and outcome after shunt surgery.

## Materials and methods

### Patients

This was a retrospective single-center study with iNPH patients enrolled from 2014 to 2018 and treated with implantation of a ventriculoperitoneal shunt system from 2014 to 2019 at Uppsala University Hospital who had given written informed consent to participate in a biobank study (Dnr 2013/278). Clinical data of the patients were collected 3 and 12 months after shunt surgery, with demographics presented in Table 1.

**Table 1:** Demographics of study participants, preoperative symptoms and outcome measured in iNPH scale

Characteristics	*n*	
Age, years, median (IQR)	74	74 (71, 78)
Sex, Female (%)	74	35 (47)
Time to follow up, months, median (IQR)	70	12 (11, 13)
**Preoperative symptoms**		
Gait, median (IQR)	73	71 (43, 86)
Balance, median (IQR)	73	67 (67, 83)
Continence, median (IQR)	70	60 (40, 80)
mRS, median (IQR)	74	3.0 (3.0, 4.0)
MMSE, median (IQR)	73	27 (25, 28)
INPH scale total, median (IQR)	74	55 (44, 64)
**Postoperative symptoms**,** follow up**		
Gait, median (IQR)	70	71 (57, 86)
Balance, median (IQR)	61	67 (67, 83)
Continence, median (IQR)	56	80 (60, 100)
mRS, median (IQR)	60	3 (3, 4)
MMSE, median (IQR)	70	27 (25, 28)
iNPH scale total, median (IQR)	68	67 (50, 81)

mRS = modified Rankin scale; MMSE = mini-mental state examination; iNPH scale total = iNPH scale without the cognitive tests

Patients were diagnosed with iNPH according to international guidelines [21]. The Swedish Ethical Review Authority approved the study (Dnr 2018/168)

### CSF samples

CSF samples were collected through lumbar puncture at the time of diagnostic work-up, preoperatively. The samples were stored in polypropylene tubes and frozen at − 70 °C. For the current analysis, the samples were thawed, divided into microtubes, and then refrozen at − 70 °C. This cohort and CSF samples was one of the cohorts included in a previous study [22] with different aims.

### Assessments of symptoms

Clinical symptoms were evaluated at the time of CSF sampling, and 3 and 12 months after shunt surgery. If a complication rendered both the 3- and 12-month follow-ups unreliable, a new follow-up visit was conducted 3 months after a reoperation and was included as the follow-up in this study.

Cognitive symptoms were graded using the mini-mental state examination (MMSE) and total symptom assessments with the iNPH scale by Hellström et al. [23]. This scale is based on grading four major symptom domains (gait, balance, continence, and cognitive function) and is used to assess a patient’s disability. Each domain is scored between 0 and 100, where 0 represents the most severe state possible and 100 is the performance of an age-matched healthy individual. A total score is calculated as the average of the four domains, with gait receiving double weight. If any domain is missing (as the cognitive domain was in this study, since it was not implemented at our center during the first part of the inclusion period), the average is calculated based on the available domains. Table 1 presents the clinical symptoms grouped into domains, along with the median scores and interquartile range (IQR) at baseline and follow up. Specially trained physiotherapists and occupational therapists performed symptom assessments.

### PEA

CSF protein levels were measured using the Olink Target 96 Inflammation panel (Olink Proteomics, Uppsala, Sweden) at SciLifeLab Affinity Proteomics (Uppsala, Sweden). The Target panels employ PEA technology [24]. Briefly, one microliter of CSF per sample was analyzed for 92 proinflammatory proteins with paired oligonucleotide-labeled antibodies that generate a unique DNA barcode upon binding to the target in close proximity. The barcode was quantified via real-time PCR using an Olink Signature instrument. Protein expression was reported as Normalized Protein eXpression (NPX), which is a relative value on a log_2_ scale, meaning that an increase of 1 NPX equals a doubling of the protein concentration. Data preprocessing, including quality control and normalization, was conducted using Olink Signature Software. All assay validation data (detection limits, intra- and inter-assay precision data, etc.) are available on the manufacturer’s website (http://www.olink.com).

### Statistics

A total of ninety-two proteins were analyzed within the neuroinflammatory spectrum; however, 32 were excluded because they did not exceed a 50% detection threshold. Missing values were imputed using the limit of detection (LOD). In this step, we used all the patient material, even though four patients did not have follow-ups.

#### Baseline symptoms and outcome analysis

Two types of linear regression models were created to examine the relationship between protein (NPX) levels and baseline symptoms and outcome. Both models were adjusted for age and sex, and the outcome models for baseline symptoms. The symptoms studied were categorized into five groups: gait, balance, continence, modified Rankin scale (mRS), MMSE, and a total calculation with all domains in the iNPH scale without cognitive symptoms (iNPH scale total). To control for multiple analyses, the Benjamini-Hochberg procedure was used to adjust the number of tested proteins, with a false discovery rate (FDR) set at 5%.

#### Classification of protein groups

The 60 proteins analyzed were further categorized into four groups based on their significant roles in the cellular function or response to neuroinflammation. These groups included the immune system, signal transduction, cellular responses to stimuli, and gene expression (transduction). Some proteins were classified into more than one group.

#### Gene set enrichment analysis (GSEA)

Based on the group categorization, it was determined whether specific sets of proteins were overrepresented in the dataset by conducting a GSEA. GSEA is a computational method that assesses whether the expression levels of a gene set differ significantly between two phenotypes [25]. An enrichment score was calculated for each protein set, and a Normalized Enrichment Score (NES) was used as a measurement variable.

## Results

### Patients

A total of 74 iNPH patients were included with available preoperative clinical data and CSF proteome analysis. Four of these were excluded from outcome analysis due to a lack of follow-up data. The reasons for exclusion were surgery performed in another part of Sweden, long travel distance to our clinic, postoperative deterioration due to unrelated medical problems, and one patient with early-stage iNPH who never underwent surgery. The outcome assessment was performed 12 months postoperatively in 68 patients and 3 months postoperatively in 2 patients.

The median age of the 74 patients was 74 years (IQR: 71–78), and 35% were women. The median follow-up time was 12 months (IQR 11,13). Table 1 presents all clinical data before and after shunt surgery.

### Association between protein levels and baseline symptoms

A linear regression model was used to assess the association between NPX (protein levels) and baseline symptoms, as measured by the three domains of the iNPH scale and the MMSE.

**Fig. 1:**
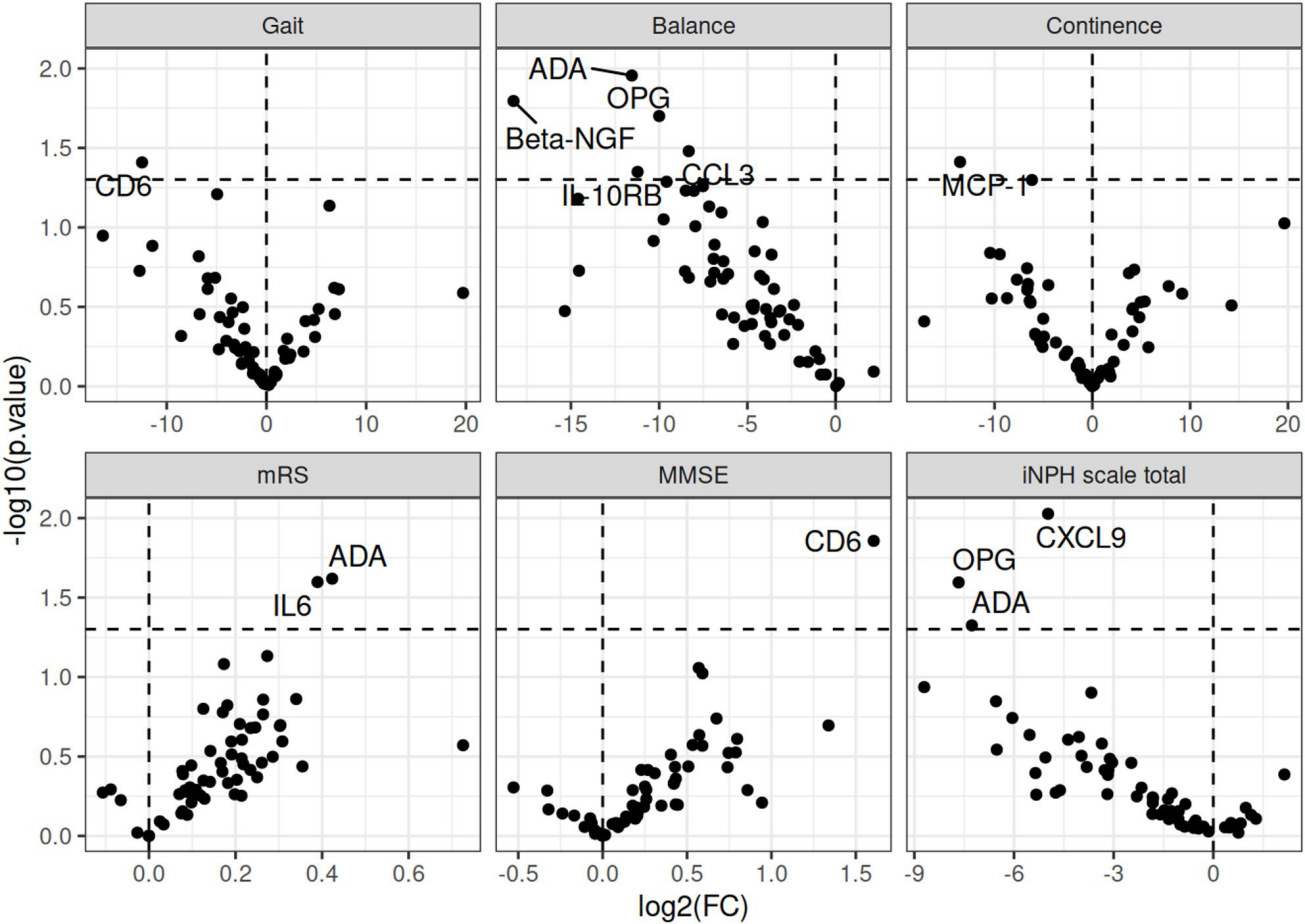
Volcano plot of association between protein level (NPX) and baseline symptoms. Adjustments made for age, sex. mRS = modified Rankin scale; MMSE = mini-mental state examination; iNPH scale total = iNPH scale without the cognitive tests

Notably, CD6 showed a negative association with gait but a positive association with MMSE scores. ADA was negatively associated with balance, but positively associated with mRS. Additionally, MCP-1 exhibited a negative association with continence, and CXCL9 was negatively associated with the total iNPH scale score. However, after correction for multiple comparisons, no associations remained statistically significant at the 5% FDR threshold (Table 2; Fig. 1).

**Table 2:** Association between protein level (NPX) and symptoms at baseline, adjusted for age and sex as computed using linear regression. Only associations with Raw *p*-values below 0.05 are shown. The *q*-value represents the adjusted p-value, corresponding to the false discovery rate (FDR) estimated by the Benjamini–Hochberg procedure. Associations with *q*-values below 0.05 (FDR < 5%) were considered statistically significant

protein	Coefficient	95% CI	*p*	*q*
**Gait**				
CD6	-12	-24.31, -0.65	0.039	0.98
**Balance**				
ADA	-12	-20.37, -2.73	0.011	0.39
Beta-NGF	-18	-33.00, -3.51	0.016	0.39
OPG	-10	-18.37, -1.63	0.020	0.39
CCL3	-8.3	-15.96, -0.69	0.033	0.39
IL-10RB	-11	-22.17, -0.27	0.045	0.39
**Continence**				
MCP-1	-14	-26.34, -0.72	0.039	0.82
**mRS**				
ADA	0.42	0.06, 0.79	0.024	0.68
IL6	0.39	0.05, 0.73	0.025	0.68
**MMSE**				
CD6	1.6	0.34, 2.88	0.014	0.83
**iNPH scale total**				
CXCL9	-5.0	-8.69, -1.26	0.0094	0.56
OPG	-7.7	-14.36, -0.97	0.025	0.76
ADA	-7.3	-14.45, -0.09	0.047	0.93

mRS = modified Rankin scale; MMSE = mini-mental state examination; iNPH scale total = iNPH scale without the cognitive tests

The association between the NPX of proteins and age and sex is represented in Supplementary Tables1 and illustrated in a volcano plot in Supplementary Fig. 1.Only a third of the proteins were significantly correlated with age.

### Association between protein levels and outcome

**Fig. 2:**
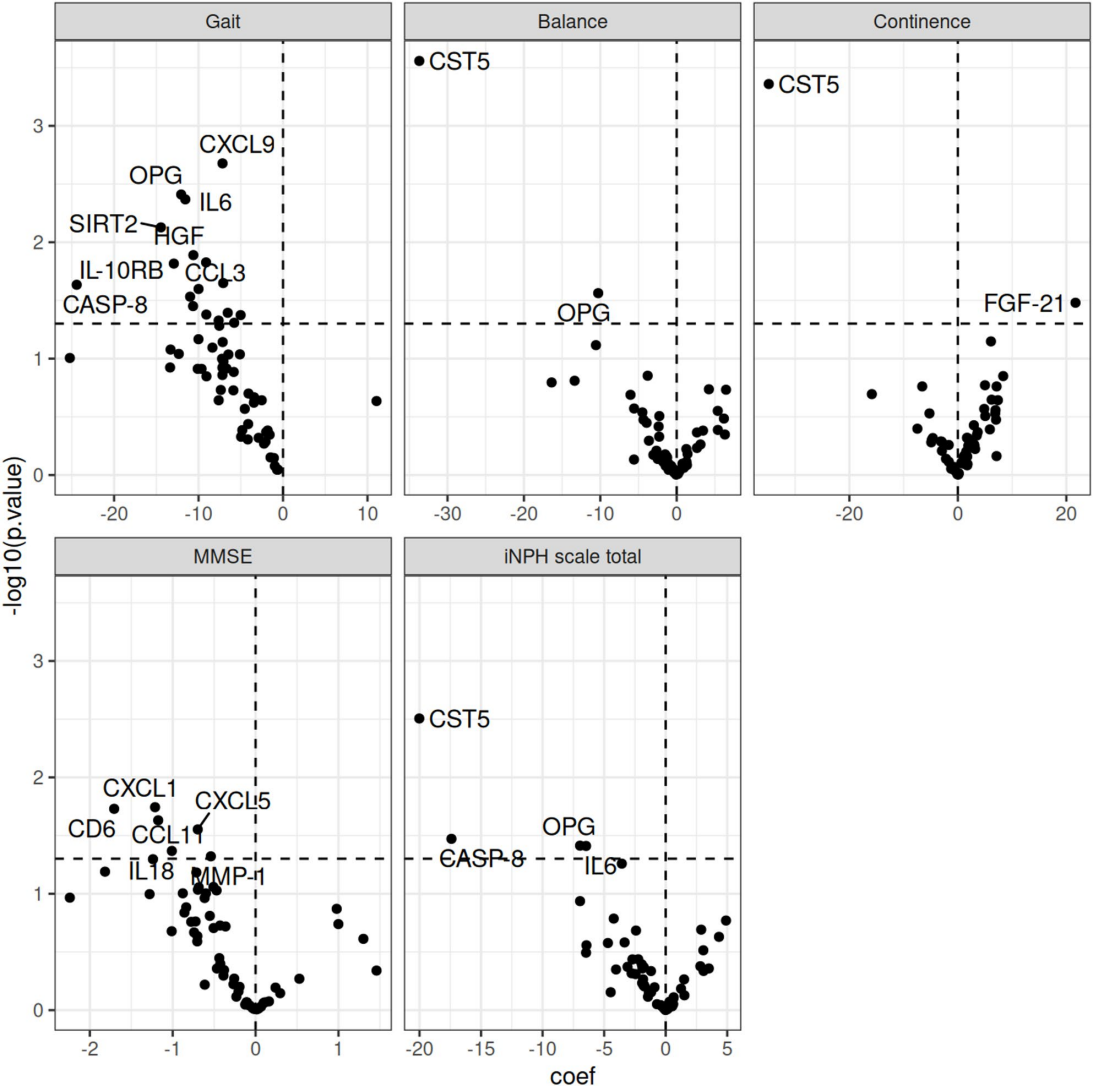
Volcano plot of association between protein level (NPX) and outcome. Adjustments were made for age, sex, and symptoms at baseline. MMSE = mini-mental state examination; iNPH scale total = iNPH scale without the cognitive tests

We analysed the correlation between NPX (protein levels) and postoperative improvement, measured as the difference between pre- and postoperative scores of the total iNPH scale, its separate domains: gait, balance, continence, as well as the MMSE. Levels of CST5 was negatively associated with improvements of balance and continence (b = -34, q = 0.017 and b = -35, q = 0.026, respectively) but not with outcome in the total iNPH-scale (b = -20, q = 0.19). There was a trend towards a negative association between improvements in gait function and levels of CXCL9, OPG, and IL-6 (q-value = 0.086), Table 3; Fig. 2.

**Table 3:** Association between protein level (NPX) and outcome, adjusted for age, sex and symptoms at baseline. Only associations with p-value below 0.05 are shown. The q-value corresponds to the false discovery rate (FDR), estimated using the Benjamini-Hochberg procedure. An FDR threshold of 5% was applied

Protein	Coefficient	95% CI	*p*	q
**Gait**				
CXCL9	-7.2	-11.63, -2.70	0.0021	0.086
OPG	-12	-20.10, -4.01	0.0039	0.086
IL6	-12	-19.37, -3.76	0.0043	0.086
SIRT2	-14	-24.91, -4.01	0.0075	0.11
HGF	-11	-18.91, -2.32	0.013	0.13
CCL3	-9.1	-16.38, -1.84	0.015	0.13
IL-10RB	-13	-23.29, -2.57	0.015	0.13
MCP-2	-7.1	-13.13, -1.03	0.022	0.15
CASP-8	-24	-45.41, -3.45	0.023	0.15
CD40	-10	-18.72, -1.28	0.025	0.15
TWEAK	-11	-20.86, -1.14	0.029	0.16
MCP-1	-11	-20.55, -0.75	0.035	0.17
CCL23	-6.5	-12.79, -0.30	0.040	0.17
TNFRSF9	-9.1	-17.81, -0.35	0.042	0.17
CCL19	-5.0	-9.86, -0.18	0.042	0.17
CX3CL1	-7.6	-15.14, -0.10	0.047	0.17
CD8A	-5.8	-11.54, -0.02	0.049	0.17
**Balance**				
CST5	-34	-51.18, -16.22	0.00028	0.017
OPG	-10	-19.37, -1.18	0.027	0.82
**Continence**				
CST5	-35	-53.57, -16.15	0.00044	0.026
FGF-21	22	1.80, 41.57	0.033	0.94
**MMSE**				
CXCL1	-1.2	-2.21, -0.22	0.018	0.36
CD6	-1.7	-3.12, -0.30	0.019	0.36
CCL11	-1.2	-2.19, -0.16	0.023	0.36
CXCL5	-0.70	-1.32, -0.08	0.028	0.36
IL18	-1.0	-1.99, -0.03	0.043	0.36
MMP-1	-0.54	-1.07, -0.01	0.048	0.36
**iNPH scale total**				
CST5	-20	-33.03, -7.0	0.0031	0.19
CASP-8	-17	-33.43, -1.38	0.034	0.58
OPG	-7.0	-13.54, -0.38	0.039	0.58
IL6	-6.5	-12.60, -0.34	0.039	0.58

MMSE = mini-mental state examination; iNPH scale total = iNPH scale without the cognitive tests

### Protein groups of different protein pathways and GSEA

Linear regression analyses were performed for four categorized protein groups based on biological pathways to explore associations with postoperative outcomes. However, no significant associations were found between these protein groups and symptom improvement, as measured by gait, balance, continence, iNPH scale total and MMSE scores. GSEA was performed based on a ranked list of all proteins, where the t-statistic determined the ranking. A normalized enrichment score (NES) was then computed for each gene set, allowing them to be ranked according to their association with the outcome. The groups of the outcome mainly showed downregulated proteins. The gait group had all proteins downregulated, while the balance group mostly downregulated, except for signal transduction proteins (CD8A, CD5) that were upregulated. The continence group also showed downregulation but had upregulated signal transduction proteins (CD8A, CD5, CD6, TGF-beta-1). The MMSE scored the highest, with upregulation of proteins related to cellular responses (SIRT2, FGF-21, FGF-5, CST5), while the iNPH scale total was mainly downregulated, Supplementary Table 2.

## Discussion

### Summary of the main findings

This study investigated associations between neuroinflammatory proteins in CSF and preoperative symptom severity and postoperative outcomes in patients with iNPH. Using a high-sensitivity PEA, we identified 60 proteins related to neuroinflammation. We assessed their relationship to clinical symptoms, as measured by the Swedish iNPH scale and the MMSE, before and after shunt surgery.

There were no associations between levels of inflammatory proteins and symptom severity before shunt surgery. Our findings align with prior studies reporting inconsistent associations between CSF biomarkers and clinical features in iNPH. The disease is likely multifactorial, involving vascular, neurodegenerative, and mechanical components that are not easily captured by a static CSF proteomic snapshot. Moreover, although mechanistically relevant, the proteins evaluated may reflect ongoing or past inflammation and were measured at a single time point, whereas symptoms in iNPH typically develop gradually over months to years.

Current evidence supporting a role of neuroinflammation in iNPH pathophysiology is limited, and our analyses identified only one protein (CST5) with a statistically significant association with improvement in iNPH symptom scales after correction for multiple testing. This association was limited to balance and continence, and not to the overall iNPH scale, and therefore should be interpreted with caution. We also observed a general non-significant trend toward negative associations between protein levels and clinical outcome, suggesting that more pronounced CNS inflammation might be unfavorable for recovery after shunting. However, pathway-based analyses (grouped protein function and GSEA) did not reveal any specific overrepresented biological processes related to clinical improvement.

The protein most strongly associated with outcome was CST5 (cystatin-D), a cysteine protease inhibitor predominantly found in saliva and tears. CST5 inhibits cathepsin S, which in the CNS contributes to extracellular matrix remodeling and barrier integrity at the choroid plexus and meninges. Interestingly, CST5 has also been shown to rise very early in blood after traumatic brain injury and has been proposed as an early inflammatory biomarker of brain damage. Although its role in iNPH has not been established, these findings suggest that CST5 could be mechanistically relevant for disease processes affecting barrier function and CSF dynamics [26–28]. In the present study, adjustment for multiple analyses was deliberately conservative, with the false discovery rate controlled at 5% to reduce the risk of Type I errors. Beyond CST5, there was a trend toward negative associations between improvements in gait function and levels of CXCL9, OPG, and IL-6. These proteins are all linked to immune activation and inflammatory signalling. CXCL9 as a T-cell–attracting chemokine, OPG as a modulator of TNF-family cytokines, and IL-6 as a pleiotropic pro-inflammatory mediator, were also identified earlier in active iNPH [29–31].

However, the sparse results of statistically significant findings limit the strength of these associations. It remains unclear whether these proteins have causal relevance, are secondary to other disease mechanisms, or reflect systemic inflammatory processes unrelated to shunt responsiveness.

The strength of this study was that all study materials were obtained from the same hospital, ensuring uniform diagnostic routines that minimized sources of error, and all CSF analyses were conducted simultaneously, ensuring solid results. Statistical analyses were conservatively adjusted for multiple analyses, adjusted for confounders, including age, sex, and symptoms at baseline. The method PEA has shown higher sensitivity and specificity than other commonly used methods [32].

Some limitations need to be considered. The conservative correction for multiple testing was used to minimize the risk of type I errors but could of course instead have induced type II errors and mask true associations, especially given the functional overlap between the proteins analysed. Although larger than some prior studies, our cohort may have been underpowered to detect small-to-moderate effect sizes across numerous proteins. We did not stratify patients by disease duration or stage, which may influence protein levels and treatment response.

Furthermore, it is essential to recognize that different comorbidities mimicking symptoms of iNPH may exist at higher age when it concerns gait, balance, continence, and cognition. For example, determining comorbid Alzheimer’s disease in iNPH patients can be challenging and can potentially negatively impact the outcome.

Our effort to classify proteins into immunological groups was challenging. We organized the proteins into four groups based on their primary functions to provide a more straightforward overview of the results. Categorizing a protein into a specific group is difficult because many proteins have multiple functions. The immunological roles of proteins in cells are interconnected, and a single protein can activate various processes. Consequently, dividing proteins into specific cellular groups may have limited usefulness.

Moreover, CSF samples were collected at a single time point. Longitudinal sampling before and after surgery could better capture dynamic changes in protein expression, which will be addressed in future studies.

## Conclusion

This study found no associations between preoperative symptom severity and levels of inflammatory proteins in CSF. CST5 emerged as the only protein significantly linked to postoperative improvement, suggesting a possible mechanistic role in iNPH pathophysiology. Larger, longitudinal studies are needed to validate these results and clarify the role of inflammatory pathways in iNPH.

## Supplementary Information

Below is the link to the electronic supplementary material.

Supplementary Material 1

## Data Availability

The datasets analyzed during the current study are available from the corresponding author on request.

## Abbreviations

BBB: Blood-brain barrier
CSF: Cerebrospinal fluid
FDR: False discovery rate
GSEA: Gene set enrichment analysis
IL: Interleukins
iNPH: Idiopathic normal pressure hydrocephalus
IQR: Interquartile range
LOD: Limit of detection
MMSE: Mini-mental state examination
mRS: Modified Rankin scale
NPX: Normalized Protein eXpression
PEA: Proximity extension assay
