# Supplementary material for: Inflammatory protein profiles and shunt response in iNPH
Source: Fluids Barriers CNS. 2026 Jan 7;23:5. doi: 10.1186/s12987-025-00751-9 (PMC12784546; doi:10.1186/s12987-025-00751-9)
Supplement: Supplementary file 1 — Supplementary Material 1 [file 12987_2025_751_MOESM1_ESM.docx]

**Supplement**

**Supplementary table 1.** Association between protein and age and sex. Sorted according to lowest p-value (age or female).

**Protein Age Female**

| **coefficient 95% CI p coefficient 95% CI p** | | | | | | |
| --- | --- | --- | --- | --- | --- | --- |
| TWEAK | 0.036 | 0.02, 0.05 | 5.0 × 10^−5^ | 0.12 | -0.09, 0.33 | 0.27 |
| PD-L1 | 0.036 | 0.02, 0.05 | 1.5 × 10^−4^ | 0.072 | -0.15, 0.30 | 0.53 |
| CDCP1 | 0.030 | 0.01, 0.05 | 2.5 × 10^−4^ | 0.013 | -0.18, 0.21 | 0.89 |
| HGF | 0.037 | 0.02, 0.06 | 2.9 × 10^−4^ | −0.037 | -0.29, 0.21 | 0.76 |
| OPG | 0.039 | 0.02, 0.06 | 3.1 × 10^−4^ | −0.35 | -0.61, -0.10 | 7.6 × 10^−3^ |
| CXCL9 | 0.067 | 0.03, 0.10 | 4.6 × 10^−4^ | 0.12 | -0.33, 0.58 | 0.59 |
| CD244 | 0.032 | 0.01, 0.05 | 6.8 × 10^−4^ | −0.17 | -0.40, 0.06 | 0.14 |
| Beta-NGF | 0.020 | 0.01, 0.03 | 7.9 × 10^−4^ | −0.023 | -0.17, 0.12 | 0.76 |
| IL-10RB | 0.027 | 0.01, 0.04 | 8.0 × 10^−4^ | −0.066 | -0.26, 0.13 | 0.50 |
| SIRT2 | 0.027 | 0.01, 0.04 | 8.8 × 10^−4^ | −0.086 | -0.28, 0.11 | 0.38 |
| LIF-R | 0.023 | 0.01, 0.04 | 9.3 × 10^−4^ | −0.089 | -0.26, 0.08 | 0.29 |
| CSF-1 | 0.023 | 0.01, 0.04 | 1.3 × 10^−3^ | −0.0055 | -0.18, 0.17 | 0.95 |
| uPA | 0.030 | 0.01, 0.05 | 1.4 × 10^−3^ | 0.062 | -0.17, 0.29 | 0.60 |
| SCF | 0.031 | 0.01, 0.05 | 2.0 × 10^−3^ | −0.17 | -0.42, 0.08 | 0.17 |
| VEGFA | 0.031 | 0.01, 0.05 | 2.3 × 10^−3^ | −0.0071 | -0.25, 0.24 | 0.95 |
| CCL23 | 0.040 | 0.01, 0.07 | 3.3 × 10^−3^ | −0.16 | -0.50, 0.17 | 0.34 |
| FGF-5 | 0.032 | 0.01, 0.05 | 3.7 × 10^−3^ | 0.0060 | -0.26, 0.28 | 0.96 |
| CD5 | 0.0074 | -0.01, 0.03 | 0.43 | 0.35 | 0.11, 0.59 | 4.2 × 10^−3^ |
| STAMPB | 0.015 | 0.00, 0.03 | 7.1 × 10^−3^ | 0.013 | -0.13, 0.15 | 0.86 |
| CD40 | 0.025 | 0.01, 0.04 | 9.1 × 10^−3^ | −0.020 | -0.26, 0.22 | 0.87 |
| CX3CL1 | 0.029 | 0.01, 0.05 | 0.010 | −0.018 | -0.30, 0.26 | 0.90 |
| DNER | 0.0073 | 0.00, 0.01 | 0.011 | −0.060 | -0.13, 0.01 | 0.092 |
| TGF-alpha | 0.017 | 0.00, 0.03 | 0.012 | −0.031 | -0.20, 0.14 | 0.72 |
| CXCL5 | 0.043 | 0.01, 0.08 | 0.013 | 0.11 | -0.32, 0.55 | 0.60 |
| CCL25 | 0.022 | 0.00, 0.04 | 0.013 | −0.24 | -0.46, -0.02 | 0.034 |
| FGF-19 | 0.033 | 0.01, 0.06 | 0.018 | −0.041 | -0.39, 0.30 | 0.81 |
| IL7 | 0.015 | 0.00, 0.03 | 0.019 | −0.026 | -0.19, 0.13 | 0.75 |
| Flt3L | 0.018 | 0.00, 0.03 | 0.021 | −0.15 | -0.34, 0.04 | 0.13 |
| CCL19 | 0.041 | 0.01, 0.08 | 0.021 | −0.50 | -0.95, -0.06 | 0.027 |
| LAP TGF-beta-1 | 0.034 | 0.01, 0.06 | 0.022 | 0.22 | -0.15, 0.60 | 0.24 |
| CD6 | 0.0014 | -0.01, 0.02 | 0.86 | 0.23 | 0.03, 0.43 | 0.028 |
| 4E-BP1 | 0.032 | 0.00, 0.06 | 0.028 | −0.037 | -0.40, 0.32 | 0.84 |
| ADA | 0.021 | 0.00, 0.04 | 0.032 | −0.12 | -0.37, 0.12 | 0.31 |
| MCP-4 | 0.042 | 0.00, 0.08 | 0.033 | 0.079 | -0.41, 0.56 | 0.75 |
| MCP-1 | 0.0026 | -0.01, 0.02 | 0.75 | −0.23 | -0.44, -0.02 | 0.034 |
| IL6 | −0.0095 | -0.03, 0.01 | 0.36 | −0.27 | -0.53, -0.01 | 0.042 |
| CXCL1 | 0.023 | 0.00, 0.04 | 0.047 | −0.11 | -0.40, 0.17 | 0.42 |
| CCL3 | 0.017 | -0.01, 0.04 | 0.14 | −0.28 | -0.56, 0.00 | 0.053 |
| OSM | 0.013 | -0.00, 0.03 | 0.056 | 0.0034 | -0.17, 0.17 | 0.97 |
| TRAIL | 0.019 | -0.00, 0.04 | 0.064 | 0.10 | -0.16, 0.36 | 0.43 |
| IL-18R1 | 0.022 | -0.00, 0.05 | 0.065 | 0.036 | -0.26, 0.34 | 0.81 |
| CCL4 | 0.020 | -0.00, 0.04 | 0.067 | −0.15 | -0.42, 0.13 | 0.30 |
| CST5 | 0.0057 | -0.00, 0.01 | 0.23 | −0.11 | -0.23, 0.01 | 0.068 |
| CXCL6 | 0.027 | -0.00, 0.06 | 0.069 | −0.19 | -0.57, 0.18 | 0.31 |
| MMP-1 | 0.037 | -0.00, 0.08 | 0.074 | 0.0017 | -0.51, 0.52 | 0.99 |
| CXCL11 | 0.014 | -0.02, 0.05 | 0.38 | 0.36 | -0.04, 0.76 | 0.076 |
| CCL11 | 0.019 | -0.00, 0.04 | 0.081 | −0.080 | -0.35, 0.19 | 0.55 |
| IL8 | 0.015 | -0.00, 0.03 | 0.13 | −0.13 | -0.38, 0.11 | 0.28 |
| MMP-10 | 0.020 | -0.01, 0.05 | 0.14 | −0.17 | -0.51, 0.16 | 0.31 |
| TNFB | 0.011 | -0.01, 0.03 | 0.24 | 0.16 | -0.07, 0.39 | 0.17 |
| CASP-8 | 0.0055 | -0.00, 0.01 | 0.17 | 0.027 | -0.07, 0.13 | 0.59 |
| IL18 | 0.015 | -0.01, 0.04 | 0.18 | −0.090 | -0.37, 0.18 | 0.51 |
| IL-12B | 0.013 | -0.01, 0.03 | 0.25 | 0.18 | -0.10, 0.45 | 0.20 |
| TNFRSF9 | 0.0070 | -0.01, 0.03 | 0.46 | −0.14 | -0.37, 0.10 | 0.26 |
| TNFSF14 | 0.0066 | -0.01, 0.02 | 0.37 | 0.090 | -0.09, 0.28 | 0.33 |
| MCP-2 | 0.012 | -0.01, 0.04 | 0.38 | −0.016 | -0.36, 0.33 | 0.93 |
| CXCL10 | 0.016 | -0.02, 0.06 | 0.44 | 0.10 | -0.41, 0.62 | 0.69 |
| CD8A | −0.0063 | -0.04, 0.02 | 0.66 | −0.13 | -0.49, 0.23 | 0.48 |
| CCL28 | 0.0038 | -0.01, 0.02 | 0.58 | −0.037 | -0.21, 0.14 | 0.68 |
| FGF-21 | −0.00079 | -0.01, 0.01 | 0.88 | −0.018 | -0.14, 0.11 | 0.78 |


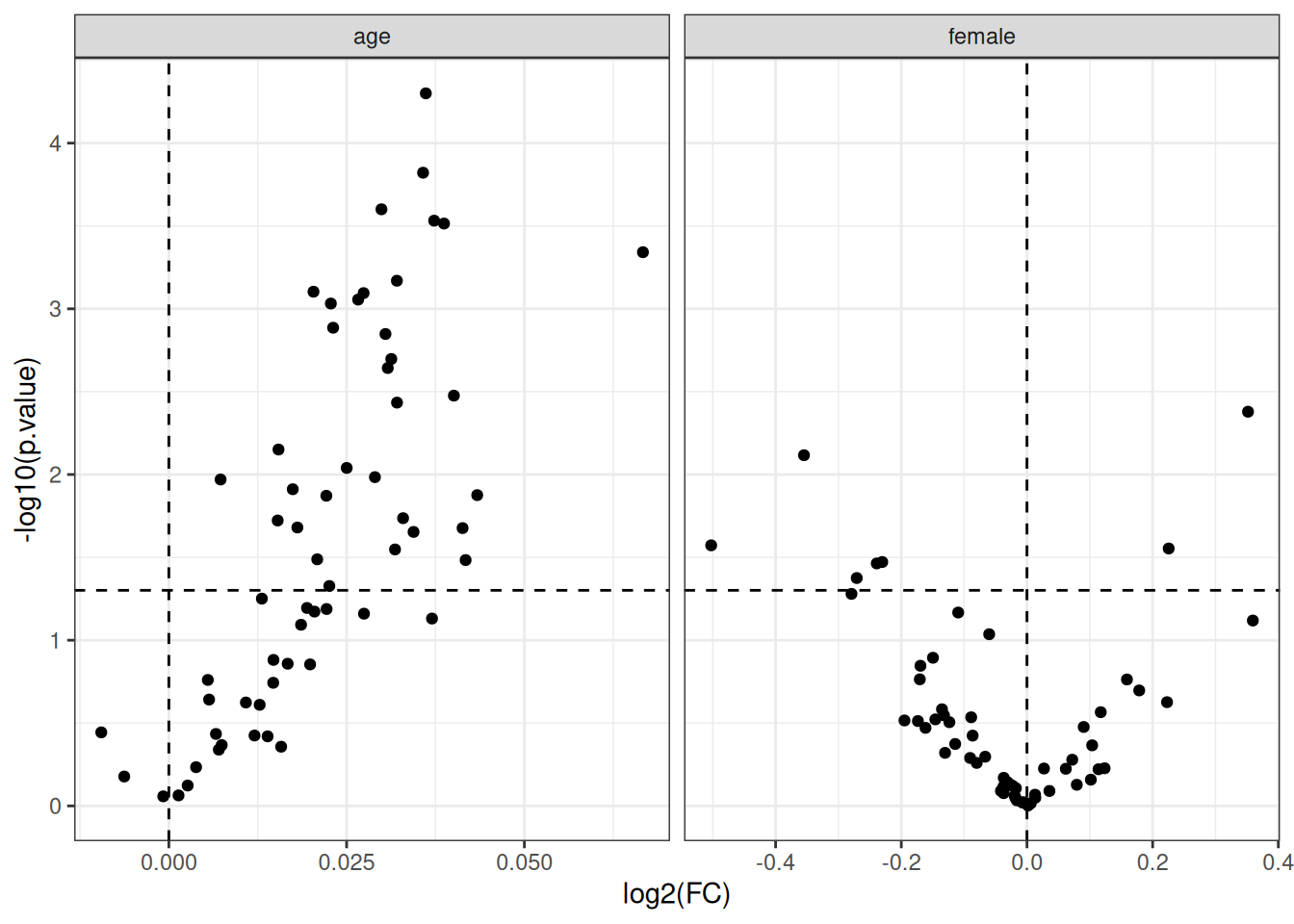


**Supplementary figure 1.** Volcano plot of association between protein level, age, and sex as computed using linear regression.

**Supplementary figure 2.** Volcano plot of association between protein level (NPX) and outcome separated in groups (colored) as computed using linear regression. The data is adjusted for age, sex and symptoms of baseline. INPH scale total is all domains without MMSE.


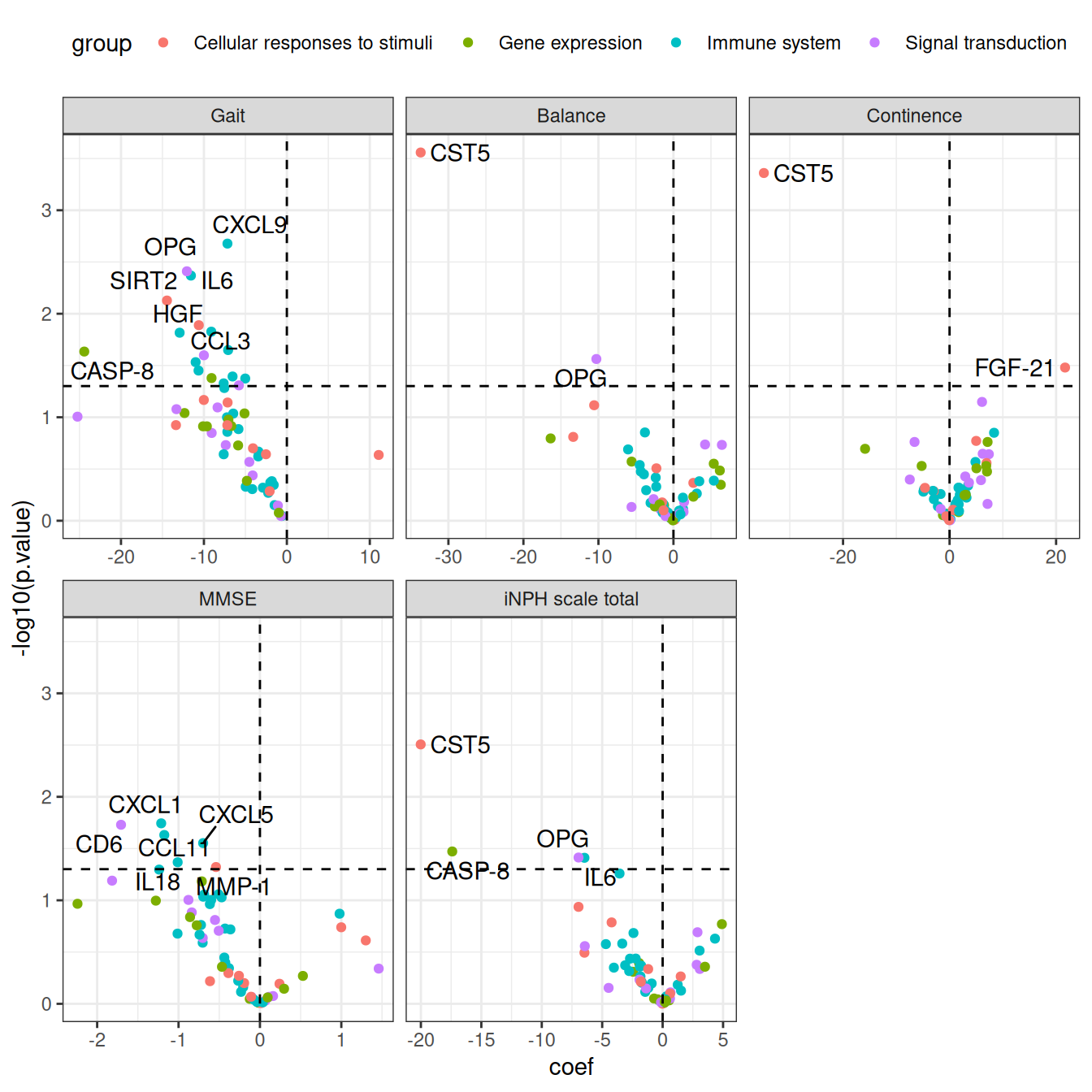


**Supplement, table 2.** Gene set enrichment analysis (GSEA) of proteins associated with follow up based on gait, balance, continence, MMSE domains and iNPH scale total. iNPH scale total is all domains without MMSE.

| \| **pathway** \| **pval** \| **padj** \| **NES** \| **size** \| **Leading Edge** \| \| --- \| --- \| --- \| --- \| --- \| --- \| \| **Gait** \|  \|  \|  \|  \|  \| \| Cellular responses to stimuli \| 0.72 \| 0.72 \| -0.81 \| 11 \| SIRT2, HGF, TWEAK, Flt3L, FGF-5, FGF-21, uPA \| \| Gene expression \| 0.47 \| 0.72 \| -1.00 \| 13 \| SIRT2, CASP-8, TNFRSF9, Beta-NGF,4E-BP1, VEGFA, SCF, LIF-R, TGF-alpha \| \| Immune system \| 0.14 \| 0.56 \| -1.31 \| 27 \| CXCL9, IL6, CCL3, IL-10RB, MCP-2, TWEAK, MCP-1, CCL23, CCL19, CXCL1, IL18 \| \| Signal transduction \| 0.57 \| 0.72 \| -0.93 \| 15 \| OPG, CD40, TWEAK, CD8A, Flt3L, PD-L1, STAMPB, DNER \| \| **Balance** \|  \|  \|  \|  \|  \| \| Cellular responses to stimuli \| 0.07 \| 0.27 \| -1.43 \| 11 \| CST5, SIRT2, FGF-21, MMP-1 \| \| Gene expression \| 0.75 \| 0.75 \| -0.80 \| 13 \| SIRT2, CASP-8, ADA \| \| Immune system \| 0.62 \| 0.75 \| -0.91 \| 27 \| CXCL9, IL8, CXCL1, IL6, CX3CL1, CCL19, CXCL6, MCP-1 \| \| Signal transduction \| 0.40 \| 0.75 \| 1.04 \| 15 \| CD8A, CD5 \| \| **Continence** \|  \|  \|  \|  \|  \| \| Cellular responses to stimuli \| 0.55 \| 0.80 \| -0.94 \| 11 \| CST5, SIRT2, FGF-5, TWEAK \| \| Gene expression \| 0.60 \| 0.80 \| 0.91 \| 13 \| TNFB, TNFSF14, VEGFA, TGF-alpha, LAP TGF-beta-1 \| \| Immune system \| 0.89 \| 0.89 \| -0.74 \| 27 \| CCL28, IL6, IL7, CXCL9, TWEAK, IL-10RB, CCL4, CD244, CX3CL1, CCL3, OSM, IL18, CCL23, CXCL5, CXCL1, CCL19, CXCL11, MCP-1, CXCL6, IL8, MCP-2, IL-18R1, MCP-4, CXCL10, CCL11 \| \| Signal transduction \| 0.20 \| 0.79 \| 1.25 \| 15 \| CD8A, CD5, CD6, Flt3L, LAP TGF-beta-1, CSF-1, TRAIL \| \| **MMSE** \|  \|  \|  \|  \|  \| \| Cellular responses to stimuli \| 0.09 \| 0.18 \| 1.33 \| 11 \| SIRT2, FGF-21, FGF-5, Flt3L, TWEAK, uPA, FGF-19, CST5, MMP-10, HGF \| \| Gene expression \| 0.62 \| 0.62 \| −0.91 \| 13 \| 4E-BP1, TNFSF14, CASP-8, TNFRSF9, LAP TGF-beta-1, SCF \| \| Immune system \| 0.08 \| 0.18 \| −1.36 \| 27 \| CXCL1, CCL11, CXCL5, IL18, CCL25, CXCL9, MCP-2, CCL23, MCP-4, CXCL11, CXCL6, IL6, CCL19, CXCL10, OSM, IL8, CD244, IL-18R1, CX3CL1 \| \| Signal transduction \| 0.38 \| 0.51 \| −1.07 \| 15 \| CD6, STAMPB, TRAIL, OPG, LAP TGF-beta-1, CD8A, OSM, CD5 \| \| **iNPH scale total** \|  \|  \|  \|  \|  \| \| Cellular responses to stimuli \| 0.26 \| 0.72 \| −1.19 \| 11 \| CST5, SIRT2, FGF-5, FGF-21, TWEAK, MMP-1, HGF, uPA \| \| Gene expression \| 0.82 \| 0.82 \| −0.73 \| 13 \| CASP-8, SIRT2 \| \| Immune system \| 0.36 \| 0.72 \| −1.08 \| 27 \| IL6, CXCL9, CCL19, IL18, IL-10RB, CCL23, CX3CL1, TWEAK, CXCL6, MCP-2, IL7, MCP-1, CCL3 \| \| Signal transduction \| 0.82 \| 0.82 \| 0.77 \| 15 \| LAP TGF-beta-1, CD5, CD6, CD8A \| |
| --- | --- | --- | --- | --- | --- | --- | --- | --- | --- | --- | --- | --- | --- | --- | --- | --- | --- | --- | --- | --- | --- | --- | --- | --- | --- | --- | --- | --- | --- | --- | --- | --- | --- | --- | --- | --- | --- | --- | --- | --- | --- | --- | --- | --- | --- | --- | --- | --- | --- | --- | --- | --- | --- | --- | --- | --- | --- | --- | --- | --- | --- | --- | --- | --- | --- | --- | --- | --- | --- | --- | --- | --- | --- | --- | --- | --- | --- | --- | --- | --- | --- | --- | --- | --- | --- | --- | --- | --- | --- | --- | --- | --- | --- | --- | --- | --- | --- | --- | --- | --- | --- | --- | --- | --- | --- | --- | --- | --- | --- | --- | --- | --- | --- | --- | --- | --- | --- | --- | --- | --- | --- | --- | --- | --- | --- | --- | --- | --- | --- | --- | --- | --- | --- | --- | --- | --- | --- | --- | --- | --- | --- | --- | --- | --- | --- | --- | --- | --- | --- | --- | --- | --- | --- | --- | --- | --- |

MMSE = mini-mental state examination; iNPH scale total = iNPH scale without the cognitive tests; Normalized Enrichment Score (NES) indicates a score for each gene set. A higher NES value reflects stronger enrichment. The gene sets are ranked according to their association with the outcome, with NES > 0 signifying upregulated genes and NES < 0 indicating downregulated genes.
